# Air pollution exposure estimation using dispersion modelling and continuous monitoring data in a prospective birth cohort study in the Netherlands

**DOI:** 10.1186/1476-069X-11-9

**Published:** 2012-02-22

**Authors:** Edith H Van den Hooven, Frank H Pierik, Sjoerd W Van Ratingen, Peter YJ Zandveld, Ernst W Meijer, Albert Hofman, Henk ME Miedema, Vincent WV Jaddoe, Yvonne De Kluizenaar

**Affiliations:** 1 The Generation R Study Group, Erasmus Medical Center, Rotterdam, The Netherlands; 2 Urban Environment and Safety, TNO, Utrecht, The Netherlands; 3 Department of Epidemiology, Erasmus Medical Center, Rotterdam, The Netherlands; 4 Department of Paediatrics, Erasmus Medical Center, Rotterdam, The Netherlands

**Keywords:** Air pollution, Dispersion modelling, Particulate matter, Nitrogen dioxide, Cohort study, Pregnant women, Prenatal development, Child health

## Abstract

Previous studies suggest that pregnant women and children are particularly vulnerable to the adverse effects of air pollution. A prospective cohort study in pregnant women and their children enables identification of the specific effects and critical periods. This paper describes the design of air pollution exposure assessment for participants of the Generation R Study, a population-based prospective cohort study from early pregnancy onwards in 9778 women in the Netherlands. Individual exposures to PM_10 _and NO_2 _levels at the home address were estimated for mothers and children, using a combination of advanced dispersion modelling and continuous monitoring data, taking into account the spatial and temporal variation in air pollution concentrations. Full residential history was considered. We observed substantial spatial and temporal variation in air pollution exposure levels. The Generation R Study provides unique possibilities to examine effects of short- and long-term air pollution exposure on various maternal and childhood outcomes and to identify potential critical windows of exposure.

## Background

Air pollution exposure has been associated with several adverse health effects, such as cardiovascular disease, respiratory disease, and total mortality [1-4]. Certain subgroups of the population, including pregnant women and their unborn children, have been suggested to be more susceptible to the adverse effects of air pollution [5,6]. Literature on the specific effects of air pollution exposure in pregnant women on outcomes such as inflammation markers, placental function, and blood pressure, is scarce. In contrast, research on the impact of air pollution exposure on birth outcomes has increased in the last decade, which has led to a number of reviews summarizing the available evidence [7,8]. Most routinely measured air pollutants (e.g., PM_10_, NO_2_, CO, O_3_, SO_2_) have been linked to increased risks of adverse birth outcomes [6]. However, results are not consistent between studies, with respect to the specific air pollutants, the relevant exposure periods, and the specific birth outcomes [7,8]. Recommendations for future research are to improve exposure assessment by incorporating detailed information on spatial and temporal patterns in air pollution concentrations and to consider a greater variety of reproductive outcomes [9]. Furthermore, it is of interest to include noise exposure data in studies on traffic-related air pollution exposure and health, since traffic is a major shared source for both air pollution and noise [10-13].

Dispersion models are applied to estimate air pollution concentrations in a study area, using data on emissions, meteorological conditions, and topography [14]. Despite the relatively costly data input, dispersion modelling is a promising method to obtain air pollution estimates for epidemiological studies, as it allows consideration of both spatial and temporal variation without the need for extensive air pollution monitoring. Dispersion models are increasingly used in combination with geographic information system (GIS) based methods. This introduces the possibility for spatial linkage of geographically referenced data, such as residential addresses, road networks, pollution sources, and street characteristics, which further enhances the quality of the modelling approach [14,15].

In this paper we describe the design of studies focused on the effects of air pollution exposure on various health outcomes in mothers and children in the Generation R Study. We describe the assessment of individual exposures to particulate matter (PM_10_) and nitrogen dioxide (NO_2_) at the home address, using a combination of continuous monitoring data and GIS based dispersion modelling techniques, taking into account both the spatial and temporal variation in air pollution. In addition, we present the distribution of exposure levels for various relevant exposure periods in the prenatal and postnatal phase, and we present exposure levels according to maternal and infant characteristics.

## Methods

### Study design

The Generation R Study is a population-based prospective cohort study from pregnancy onwards, which was designed to identify early environmental and genetic causes of normal and abnormal growth, development, and health during fetal life, childhood and adulthood. It has been described previously in detail [16,17]. In brief, the cohort includes mothers and children of different ethnicities living in the city of Rotterdam, the Netherlands. Enrolment was aimed in early pregnancy (gestational age < 18 weeks), but was allowed until the birth of the child. Out of the total number of eligible children in the study area, 61 percent participated in the study at birth. In total, 9778 mothers with a delivery date between April 2002 and January 2006 were enrolled in the study. Extensive assessments have been carried out in mothers and fathers and are currently performed in their children, who form a prenatally recruited birth cohort that will be followed until young adulthood. Data collection included questionnaires, detailed physical and ultrasound examinations, behavioural observations, and biological samples. Assessments in pregnancy were performed in each trimester. Assessments in the children in the preschool period (birth to age of 4 years) included a home-visit, questionnaires, and visits to the routine child health centres. From the age of 5 years onward, regular detailed hands on assessments are performed in all children and their parents in a research center. The study protocol was approved by the Medical Ethical Committee of Erasmus Medical Center, Rotterdam. Written informed consent was obtained from all participants.

### Air pollution exposure assessment

Individual exposures to PM_10 _and NO_2 _levels during pregnancy were assessed at the home address, using advanced spatiotemporal dispersion modelling techniques in combination with hourly air pollution measurements at three continuous monitoring sites. The exposure assessment procedure has been described previously [18,19]. Below, we give a brief summary of the procedure, including some revised information that better describes the individual steps.

#### Spatial pattern

Annual average concentrations of PM_10 _and NO_2 _for the years 2001-2008 were assessed for all addresses in the study area, using GIS and the three Dutch national standard methods for air quality modelling (considering intra-urban road traffic, traffic on highways, and industrial and other point sources) [20]. Subsequently, in order to obtain spatiotemporal patterns, spatially resolved annual concentrations were calculated for eight different wind conditions, resulting in an averaged spatially resolved concentration pattern for each wind class. Various input data was taken into account in the calculations as described earlier [18,19], including annual data on traffic intensities and annual emissions from traffic, shipping, industry, and households. The traffic intensity data was supplied by the DCMR Environmental Protection Agency Rijnmond (DCMR), and emission sources and emission data were obtained from the National Institute for Public Health and the Environment (RIVM) and the DCMR. Hourly meteorological data was obtained from observations at Rotterdam The Hague Airport, performed by the Royal Netherlands Meteorological Institute (KNMI).

#### Temporal pattern

To account for temporal variation due to different wind conditions, for each hour we derived the corresponding spatial distribution for the prevailing wind direction and wind speed at that specific hour, by means of interpolation between the eight characteristic spatial distributions. Subsequently, the spatial distributions that corresponded to the hourly wind conditions were adjusted for fixed temporal patterns of source activities. In this way, we accounted for temporal fluctuations in the contribution of air pollution sources during the month, week (e.g., working days and weekend days), and day (e.g., morning and evening rush hour). The adjustment for temporal patterns was performed for traffic and for household emissions. Traffic is the source with the strongest fluctuations in emissions within 24 hours. This 24 h-pattern is fairly stable for working days and weekend days. Hence, the contribution of traffic was scaled using an average hourly traffic intensity pattern (based on traffic counts), thereby deriving hourly intensities. We also considered the time dependence of household emissions, by applying a 24 h-pattern, and we applied a function for outdoor temperature dependence to account for seasonal fluctuations. These functions were derived from energy use statistics. In this way, hourly household emissions were estimated from annual household emissions. Emissions from industrial sources do not contribute significantly to small-scale variations in air pollution concentrations. Emissions from shipping are quite stable over time and also display relatively small temporal fluctuations. Therefore, these emissions were not adjusted for fixed temporal patterns. Nevertheless, even if some small-scale variations had occurred as a result of these emissions, the difference would have been corrected for in the next step (adjustment for hourly background concentrations).

#### Adjustment for background concentrations

The modelled hourly concentrations were adjusted for background concentrations (see also [18,19]), in order to consider the temporal fluctuations in background concentrations. This was done using continuous hourly monitoring data from three monitoring stations in the study area. The measured air pollution concentrations at these stations are considered as the sum of the background concentration and the contribution from local emission sources. We modelled the contribution of local emission sources to the PM_10 _and NO_2 _concentrations at the three monitoring stations. Subsequently, we subtracted the hourly modelled contributions from the hourly measured concentrations at the stations, thereby deriving an hourly estimate for the background concentrations. The hourly estimates for the background concentrations at the three stations were averaged, which yielded an average hourly background concentration for the study area. In the adjustment procedure, this average hourly background concentration was added to the modelled hourly contributions at the home addresses, in order to take into account the background concentration.

Continuous air pollution monitoring data was provided by DCMR. Missing values for PM_10 _concentrations at the three monitoring stations were imputed, as described earlier [18,19].

#### Modelling performance

As described above, the first step in our modelling procedure involved the assessment of annual average PM_10 _and NO_2 _concentrations, using a combination of the three Dutch standard methods. The performance of this modelling procedure based on (a combination of) the three standard methods has been evaluated by two previous studies in the same study area. These studies reported a good agreement between predicted annual average PM_10 _and NO_2 _concentrations and concentrations measured at monitoring stations [21,22]. Our dispersion modelling approach, resulting in hourly average concentrations, is a refinement of this former modelling procedure. An additional validation study of this refined modelling procedure was not feasible within the scope of this project.

#### Exposure assignment

Derived from the hourly concentrations of PM_10 _and NO_2_, we constructed a database containing daily averages (24 h) for every address, for the years 2001-2008. Allowing for residential mobility, air pollution exposure estimates were linked to the different home addresses of the participants throughout the study period. This yields a database with individual exposures, which can be used to derive average exposure estimates for any period between 2001 and 2008, depending on the specific research question. For the present paper, we describe air pollution exposure estimates for a number of pregnancy and childhood periods, to illustrate the distribution of exposure levels in participants in these potential sensitive periods. More specifically, we derived exposures for the following periods: first trimester, second trimester, third trimester, total pregnancy, birth until 6 months postnatally, and 7 until 12 months postnatally. Exposures were only calculated for periods with less than 25% of the daily averages missing. For the other periods, air pollution exposures were set to missing.

### Statistical analyses

Descriptive analyses were performed for all air pollution exposure averages, including the evaluation of the Pearson correlation coefficients between the different exposure averages. In addition, we examined mean maternal PM_10 _and NO_2 _exposure levels during total pregnancy according to maternal characteristics and infant characteristics. Information on these characteristics was obtained from questionnaires in pregnancy and from medical records, as described elsewhere [16,18]. Maternal noise exposure (based on the home address at time of delivery) was assessed in accordance with requirements of the EU Environmental Noise Directive, which has been described previously [10,16,18,23]. Information on average neigbourhood income was obtained from Statistics Netherlands as neighbourhoods' average disposable income per income receiver in the year 2004, and classified into: low (< 1400 euro/month), moderate (1400-2200 euro/month), and high (> 2200 euro/month). Season of conception and season of birth were categorized as winter (December to February), spring (March to May), summer (June to August), and fall (September to November). For all maternal and infant characteristics, we performed a one-way ANOVA followed by Bonferroni's post hoc comparison tests to examine the differences in mean air pollution exposure levels compared with the reference group. All statistical analyses were performed using PASW version 17.0 for Windows (PASW Inc., Chicago, IL).

## Results

### Air pollution exposure in the study cohort

Of the 9778 women, exposure estimates could not be calculated for 149 mothers because they had an abortion (n = 29) or intrauterine death (n = 75), or were lost-to-follow up (n = 45), and consequently no information was available on the date of conception and delivery. For the remaining 9629 women (and their 9748 children), 12188 addresses were available for the time period presented here (conception until the first year postnatally). Of all women, 74% did not move in this period, 25% changed residence once, and less than 1% moved two or three times. Of the 12188 addresses, 10518 (86%) could be linked to the air pollution exposure database, and 1938 addresses could not be linked. This was either due to missing address information, incompatible street number suffices, or to addresses situated outside of the study area of the Generation R Study [16]. As a result, air pollution exposure estimates for the present paper were available for 8810 mothers and 8921 children.

Table 1 presents the distribution of maternal PM_10 _and NO_2 _levels for a number of illustrative prenatal and postnatal periods. The number of participants with available exposure data varied for the specific periods. On average, PM_10 _and NO_2 _exposure levels during first trimester were higher than during second and third trimester, and postnatal exposure levels were lower than prenatal exposure levels. This can be explained by the decreasing trend in air pollution levels throughout the study period. Mean air pollution exposure levels during pregnancy were 30.2 μg/m^3 ^(range 23.1 to 39.9) for PM_10 _and 39.7 μg/m^3 ^(range 25.3 to 56.9) for NO_2 _(Table 1). On average, these levels are below the European Union annual limit values (40 μg/m^3 ^for both PM_10 _and NO_2_) that are defined for protection of human health [24], but a substantial proportion of the women was exposed to levels higher than these limit values. Moreover, it has been acknowledged that significant health effects may occur even below the current limit values [25].

**Table 1:** Distribution of maternal PM_10 _and NO_2 _exposure levels for different prenatal and postnatal periods

	N	Minimum	25th percentile	Mean	Median	75th percentile	Maximum
**PM_10 _exposure (μg/m^3^)**							
**Prenatal**							
First trimester	7894	22.0	27.7	30.6	30.5	33.4	43.1
Second trimester	8311	21.3	26.2	30.1	29.5	33.3	45.6
Third trimester	8438	22.0	26.6	29.8	29.8	32.0	43.5
Total pregnancy	7877	23.1	27.7	30.2	29.9	32.8	39.9
**Postnatal**							
Month 0-6	8381	22.7	27.3	29.5	29.3	31.4	39.9
Month 7-12	8082	22.8	27.0	28.8	28.7	30.5	39.3

**NO_2 _exposure (μg/m^3^)**							
**Prenatal**							
First trimester	7893	21.4	36.9	40.2	40.6	43.5	58.5
Second trimester	8310	20.2	35.2	39.6	40.5	43.9	59.7
Third trimester	8434	21.3	35.4	39.3	39.9	43.2	58.8
Total pregnancy	7889	25.3	37.0	39.7	39.5	42.2	56.9
**Postnatal**							
Month 0-6	8389	24.2	36.3	39.4	39.5	42.5	59.3
Month 7-12	8082	24.1	35.5	38.6	38.6	41.6	58.0

Air pollution exposure was estimated for different prenatal and postnatal periods: first trimester (0-18 weeks), second trimester (18-25 weeks), third trimester (25 weeks-delivery), total pregnancy, month 0-6 postnatally, and month 7-12 postnatally

Epidemiological studies often evaluate associations for air pollution exposure levels in different periods, in order to examine the relevant exposure periods, which is informative only if the correlations among these exposure levels are not too high. Table 2 shows that Pearson correlation coefficients between the different air pollution exposure averages for the present paper varied between 0.02 and 0.83. Correlations among exposure averages for the first, second, and third trimester were moderate (PM_10_: r = 0.31 to 0.48, NO_2_: r = 0.17 to 0.48). Correlations between exposure averages for the separate trimesters with exposure averages for the total pregnancy period were higher (PM_10_: r = 0.73 to 0.83, NO2: r = 0.43 to 0.51). Correlations between prenatal and postnatal exposure averages were low for PM_10 _(r = 0.13 to 0.29), and somewhat higher for NO_2 _(r = 0.22 to 0.78). PM_10 _and NO_2 _exposures averages for the same period were moderately correlated (r = 0.58 to 0.66).

**Table 2:** Correlation coefficients between period-specific PM_10 _and NO_2 _exposure averages

	PM_10_								NO_2_			
	**First trimester**	**Second trimester**	**Third trimester**	**Total pregnancy**	**Month0-6 postnatally**	**Month 7-12 postnatally**	**First trimester**	**Second trimester**	**Third trimester**	**Total pregna ncy**	**Month 0-6 postnatally**	**Month 7-12 postnatally**

**PM_10_**												

First trimester	1											

Second trimester	0.48	1										

Third trimester	0.31	0.46	1									

Total pregnancy	0.83	0.74	0.73	1								

Month 0-6 postnatally	0.19	0.13	0.34	0.29	1							

Month 7-12 postnatally	0.11	0.02	0.01	0.06	0.21	1						

**NO_2_**												

First trimester	0.59	0.36	0.19	0.51	0.28	0.01	1					

Second trimester	0.26	0.58	0.41	0.48	0.15	0.24	0.45	1				

Third trimester	0.17	0.24	0.63	0.43	0.25	0.36	0.17	0.48	1			

Total pregnancy	0.49	0.47	0.53	0.64	0.32	0.26	0.77	0.76	0.73	1		

Month 0-6 postnatally	0.48	0.21	0.22	0.42	0.66	0.27	0.66	0.22	0.30	0.57	1	

Month 7-12 postnatally	0.17	0.29	0.44	0.37	0.26	0.63	0.34	0.68	0.78	0.77	0.39	1

Values reflect Pearson correlation coefficients between air pollution exposure estimates for different prenatal and postnatal periods

**Figure 1:**
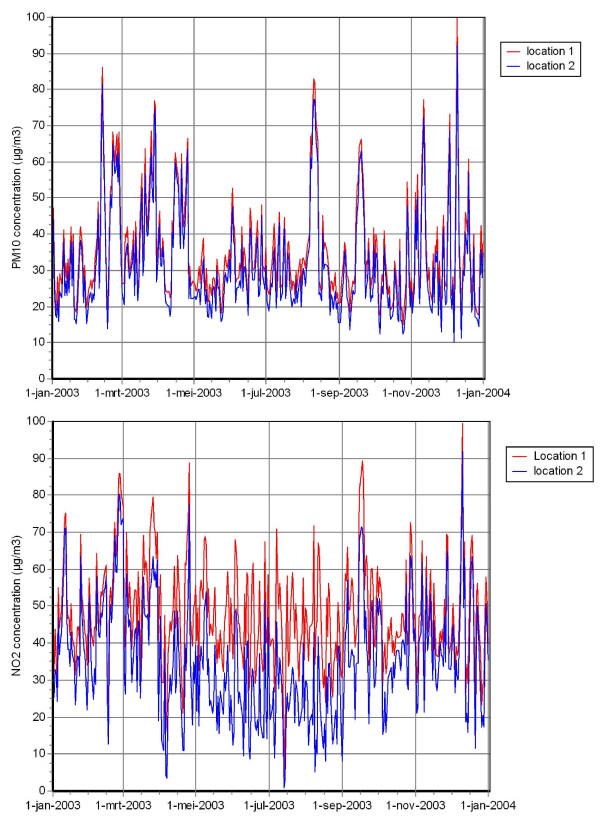
**Illustration of the temporal variation in of PM_10 _and NO_2 _exposure levels in the study area**. a. PM_10 _concentration. b. NO_2 _concentration. Estimated PM_10 _and NO_2 _concentrations in 2003 at two different locations in the study area. Location 1 is located in the city center, whereas location 2 is situated in a suburb of Rotterdam.

There was substantial spatial and temporal variation in air pollution exposure levels. We have previously published maps of the spatial distribution of annual PM_10 _and NO_2 _concentrations in the study area [18,19], which demonstrated differences in annual average concentrations up to 4-8 μg/m^3 ^between urban and suburban areas. Figure 1 presents the temporal variation in PM_10 _and NO_2 _exposure levels estimated at two different locations in the study area (one situated in the city center and one situated in a suburb of Rotterdam). Especially for NO_2_, substantial differences were observed between the two locations.

For illustrative purposes, we present mean maternal air pollution exposure during total pregnancy according to maternal characteristics (Table 3) and infant characteristics (Table 4). Table 3 shows that PM_10 _and NO_2 _exposure levels were higher for mothers who were younger than 25 years, of non-Dutch ethnicity, nulliparous, were exposed to higher noise levels, lived in a low neighbourhood income area, and whose conception occurred in summer or fall. In addition, NO_2 _exposure was slightly higher in women who continued smoking, and PM_10 _exposure was higher in women who continued to consume alcohol during pregnancy. There was a clear decrease in air pollution exposure over time: women whose conception fell between 2001 and 2003 were exposed to higher PM_10 _and NO_2 _levels during pregnancy than women with a conception date in 2004 or 2005. Table 4 shows that mothers were exposed to higher PM_10 _and NO_2 _levels when they gave birth in spring or summer, compared with winter or fall. Mean exposure levels according to the year of birth also showed a decreasing trend in air pollution concentrations between 2002 and 2006.

**Table 3:** Maternal air pollution exposure during pregnancy according to maternal characteristics

	N	**PM_10 _exposure **(μg/m^3^) Mean (SD)	**NO_2 _exposure **(μg/m^3^) Mean (SD)
**Maternal characteristics**			

**Age**			

< 25 years	1446	30.5 (3.2) *	40.4 (3.8) *

*25-30 years (Reference)*	2051	30.2 (3.1)	39.8 (4.2)

30-35 years	2998	30.1 (3.2)	39.5 (4.4) *

> 35 years	1395	30.0 (3.2)	39.5 (4.3)

**Body mass index**			

< 20 kg/m^2^	627	30.5 (3.2)	40.3 (4.2)

*20-25 kg/m^2 ^(Reference)*	3714	30.3 (3.2)	39.8 (4.2)

25-30 kg/m^2^	1843	30.3 (3.1)	39.8 (4.1)

> 30 kg/m^2^	972	30.0 (3.2)	39.6 (4.0)

Missing	734	29.1 (3.1) **	38.6 (4.7) **

**Ethnicity**			

*Dutch/Caucasian (Reference)*	4268	30.1 (3.2)	39.4 (4.5)

Turkish	622	30.1 (3.0)	40.2 (3.5) **

Moroccan	489	30.2 (3.0)	40.1 (3.5) *

Surinamese	619	30.6 (3.2) *	40.2 (4.0) **

Other	1151	30.4 (3.3) *	40.3 (4.1) **

Missing	741	29.8 (3.0)	40.1 (4.0) **

**Educational level**			

No education/primary	757	30.3 (3.1)	40.0 (3.6)

Secondary	3102	30.3 (3.2)	39.7 (4.3)

*Higher (Reference)*	3132	30.1 (3.2)	39.6 (4.4)

Missing	899	29.8 (3.0)	40.1 (4.0) *

**Parity**			

*Nulliparous (Reference)*	4129	30.3 (3.2)	40.0 (4.3)

Multiparous	3528	30.1 (3.1) *	39.5 (4.1) **

Missing	233	29.4 (3.1) **	38.8 (4.5) **

**Smoking in pregnancy**			

*No (Reference)*	4616	30.2 (3.2)	39.7 (4.2)

First trimester only	527	30.5 (3.3)	40.1 (4.6)

Continued	1059	30.5 (3.2)	40.2 (4.2) *

Missing	1688	29.6 (2.9) **	39.5 (4.2)

**Alcohol use in pregnancy**			

*No (Reference)*	3022	30.2 (3.2)	39.8 (4.1)

First trimester only	820	30.2 (3.2)	39.6 (4.4)

Continued	2415	30.4 (3.2) *	39.9 (4.3)

Missing	1633	29.7 (2.9) **	39.5 (4.2)

**Noise exposure**			

< 50 dB(A)	2985	29.6 (3.0) **	37.9 (3.3) **

*50-65 dB(A) (Reference)*	4016	30.2 (3.1)	39.8 (3.6)

> 65 dB(A)	791	32.2 (3.5) **	46.0 (4.3) **

Missing	91	29.8 (3.1)	40.0 (4.0)

**Neighbourhood income**			

Low	1141	30.9 (2.9) **	41.0 (3.2) **

*Moderate (Reference)*	4678	30.0 (3.1)	39.6 (4.2)

High	1945	30.2 (3.2)	39.6 (4.5)

Missing	126	28.4 (3.2) **	35.2 (5.5) **

**Season of conception**			

*Winter (Reference)*	2184	29.9 (3.8)	38.8 (4.5)

Spring	1850	29.7 (2.6)	38.9 (4.1)

Summer	1810	30.5 (2.4) **	41.1 (3.8) **

Fall	2046	30.5 (3.4) **	40.3 (3.9) **

**Year of conception**			

*2001 (Reference)*	345	34.6 (1.3)	39.6 (3.4)

2002	2161	33.1 (1.6) **	41.8 (3.8)

2003	2468	29.5 (3.0) **	39.9 (4.2) **

2004	2460	28.0 (2.0) **	38.2 (3.9) **

2005	456	28.4 (1.2) **	37.4 (4.1) **

** *P *< 0.01

* *P *< 0.05

Values are mean PM_10 _and NO_2 _exposure levels for the total pregnancy period. *P*-values are based on One-way ANOVA followed by Bonferroni's post hoc comparison tests to examine the differences in means compared with the Reference group

**Table 4:** Maternal air pollution exposure during total pregnancy according to infant characteristics

	N	PM_10 _exposure (μg/m^3^) Mean (SD)	NO_2 _exposure (μg/m^3^) Mean (SD)
**Child characteristics**			

**Gestational age at birth**			

< 37 weeks	463	30.4 (3.3)	40.0 (4.5)

*37-42 weeks (Reference)*	6871	30.2 (3.1)	39.7 (4.2)

< 42 weeks	556	30.1 (3.3)	39.7 (4.1)

**Birth weight**			

< 2500 grams	359	30.4 (3.1)	40.0 (4.4)

*2500-4500 grams (Reference)*	7194	30.2 (3.2)	39.7 (4.2)

> 4500 grams	337	30.0 (3.2)	39.6 (4.3)

**Season of birth**			

*Winter (Reference)*	1856	29.7 (2.7)	38.9 (4.1)

Spring	1781	30.4 (2.3) **	41.0 (3.8) **

Summer	2098	30.5 (3.4) **	40.4 (4.0) **

Fall	2155	30.0 (3.8)	38.7 (4.5)

**Year of birth**			

*2002 (Reference)*	696	33.6 (1.7)	39.6 (3.5)

2003	2406	33.2 (1.6) **	41.9 (3.9) **

2004	2548	27.6 (2.4) **	39.0 (4.2) *

2005	2214	28.8 (1.5) **	38.3 (3.9) **

2006	26	27.8 (1.3) **	36.8 (4.1) *

** *P *< 0.01

* *P *< 0.05

Values are mean PM_10 _and NO_2 _exposure levels for the total pregnancy period. *P*-values are based on One-way ANOVA followed by Bonferroni's post hoc comparison tests to examine the differences in means compared with the Reference group

## Discussion

For the participants of this large population-based cohort study, we assessed individual air pollution exposure at the home address using advanced state-of-the-art methods. By using a combination of GIS based dispersion modelling and continuous monitoring data, we were able to take into account the spatial and temporal variation in air pollution concentrations. The individual exposure estimates can be used in further epidemiological studies that examine air pollution effects in this population of mothers and children.

### Air pollution exposure

In our air pollution exposure assessment procedure, we were able to consider fine spatial and temporal contrasts in exposure by using a combination of dispersion modelling and continuous monitoring. The high temporal resolution enables investigation of relatively short exposure windows (e.g., total pregnancy, trimesters, or months) that are particularly of interest in pregnant women and children. It also facilitates identification of critical windows of exposure. These short-term exposure windows cannot be examined in studies with only annual average concentrations. In examination of the different exposure windows, the (possibly) moderate to high correlations among some of the exposure averages need to be considered when interpreting the results. Next to a high temporal resolution, detailed information on spatial contrasts in air pollution exposure is required, since ambient air pollutants display significant small-scale spatial variation. This intra-urban spatial variation has been documented especially for traffic-related pollutants such as NO_2_, black smoke, elemental carbon, ultrafine particles, and to a lesser extent for PM_10 _and PM_2.5 _[26,27]. Our exposure estimates have been used in three previous studies on air pollution effects in the same population, which suggest that exposure to air pollution during pregnancy may affect maternal and fetal health [18,19,28].

We explored whether air pollution exposure levels were differentially distributed according to maternal and infant characteristics. Associations between air pollution exposure and health may be subject to confounding, if sociodemographic and lifestyle-related factors are associated both with air pollution exposure and with health. Our illustrative findings suggest that in our cohort, air pollution exposure may be differentially distributed according to age, ethnicity, parity, neighbourhood income area, smoking, and alcohol consumption. This stresses the importance to account for these factors when analyzing the associations between air pollution exposure and health.

Rotterdam is the second largest city in the Netherlands with a high population density and the largest port of Europe. It is characterized by high emissions from road traffic, shipping, households, and industry. A few recent European studies assessed air pollution exposure in pregnant women using land-use regression modelling approaches that also considered spatiotemporal variation in exposure [29-32]. In these studies, mean NO_2 _exposure levels estimated for the entire pregnancy were slightly lower than those obtained in our cohort (i.e., around 36-37 μg/m^3 ^compared with 40 μg/m^3 ^in our cohort). None of the studies assessed PM_10 _exposure. The differences in exposure levels can be explained by various factors, including the geographic location and urbanization degree of the study area, study period (season and year), modelling approach input data, climate, meteorological conditions, and pollution sources.

Traffic-related air pollution is a complex mixture of several pollutants. We assessed exposure to PM_10 _and NO_2 _in our cohort, because these pollutants have been routinely measured in the National Air Quality Monitoring Network during the study period, and they often exceed the air quality standards at locations near heavy traffic. Furthermore, PM_10 _and NO_2 _can be regarded as markers for the traffic-related air pollution mixture and have been associated with several adverse health effects [1,2,9,33-35]. Other components that may be relevant for health (PM_2.5_, black smoke) have not been monitored during the study period and could therefore not be assessed. Up to now, we have assessed air pollution exposure until the year 2008, and we are planning to update this data for future years when the relevant monitoring data will be available (for PM_10_, NO_2_, and specific components). In addition, exposure to other, 'criteria' air pollutants such as SO_2 _and CO could be estimated in the future using the same modelling procedure.

Assigning exposures based on the home address at time of delivery may introduce exposure misclassification as a number of women change their address during pregnancy [36], and are thus exposed to different levels of air pollution. We obtained full residential history of the participants, which showed that 26% of the women moved at least once in the period between conception and the first year postnatally. Air pollution exposure estimates were assessed for the different prenatal and postnatal addresses. There can still be non-differential misclassification of air pollution exposure, since exposure levels were estimated at the home address, and people do not spend all of their time at home. Indoor, occupational, or commuting sources of air pollution have not been captured in our modelling procedures. The extent of the possible misclassification may be minor in this specific population, as pregnant women are likely to spend more time at home than non-pregnant individuals, especially in the last stage of pregnancy [37].

There is increasing awareness of the importance to incorporate information on noise exposure in studies on traffic-related air pollution exposure and health [10-13]. Thus far, few studies have included both air pollution and noise when investigating health outcomes [10,38-40]. In our previous studies on air pollution and pregnancy outcomes, we included information on noise exposure, in order to adjust for its potential confounding effect [18,19].

## Conclusions

Detailed air pollution exposure levels are available for mothers, fathers, and children in the Generation R Study and efforts are ongoing to update these exposures. The individual exposure estimates can be used in further epidemiological studies focused on the effects of prenatal and postnatal air pollution exposure on various health outcomes in mothers and children, including reproductive outcomes, growth and development, cognitive function, respiratory function, and cardiovascular outcomes. The combination with other detailed data (noise levels, biomarkers, and genetics) enables in-depth investigations and identification of critical windows of exposure.

## Abbreviations

EU: European Union; GIS: Geographic information system; PM_10: _Particulate matter with an aerodynamic diameter < 10 μm; PM_2.5: _Particulate matter with an aerodynamic diameter < 2.5 μm; NO_2: _Nitrogen dioxide; CO: Carbon monoxide; O_3: _Ozone; SO_2: _Sulfur dioxide.

## Competing interests

The authors declare that they have no competing interests.

## Authors' contributions

All authors have made substantial contribution to this study and to the writing and editing of the manuscript. Additional contributions are as follows: EHH was involved in the planning of the study, data collection, descriptive analyses, and interpretation of data, and drafted the manuscript; FHP, VWVJ and YK contributed to the design of the study, supervision, interpretation of data and critical review of the manuscript; SWR, PYJZ, and EWM designed the exposure assessment and performed exposure calculations; AH conceptionalised the Generation R study and participated in its design and conduction; HMEM contributed to the design of the study and had critical input. All authors read and approved the final manuscript.
